# Smart Materials for Environmental Remediation Based on Two-Component Gels: Room-Temperature-Phase-Selective Gelation for the Removal of Organic Pollutants Including Nitrobenzene/O-Dichlorobenzene, and Dye Molecules from the Wastewater

**DOI:** 10.1186/s11671-019-2865-6

**Published:** 2019-02-01

**Authors:** Jing Zhang, Jiahui Liu, Ciqing Tong, Shipeng Chen, Baohao Zhang, Bao Zhang, Jian Song

**Affiliations:** 1 School of Chemical Engineering and Technology, Tianjin University, Tianjin, 300350 China; 2 The Co-Innovation Center of Chemistry and Chemical Engineering of Tianjin, Tianjin, 300072 China; 3 Renai College of Tianjin University, Tianjin, 301636 China; 4 Max Planck Institute for Polymer Research, Ackermannweg 10, 55128 Mainz, Germany; 5 Department of Supramolecular and Biomaterials Chemistry, Leiden Institute of Chemistry, Leiden University, P.O. Box 9502, 2300 RA Leiden, The Netherlands

**Keywords:** Room-phase-selective-gelator (RPSG), Self-healing, Dye removal

## Abstract

Novel two-component gel systems based on aliphatic acid–hydroxy/base interaction were developed as smart materials for environmental remediation. The *G1-A16* gelator could be used directly as a powder form to selectively gel aromatic solvents (nitrobenzene and o-dichlorobenzene) from their mixtures with wastewater (containing 0.5 M sodium nitrate and 0.5 M sodium sulfate) via a simple shaking strategy at room temperature without employing co-solvents and a heating–cooling process. Meanwhile, the two-component gel system can efficiently remove the toxic dyes from the aqueous solution. The dominant factors that drive gelation in the case of the gelator and nitrobenzene or water have been studied using FT-IR, ^1^H NMR, and XRD. Overall, our research provides an efficient two-component approach for facilely tuning the properties of one-component gel for the realization of high-performance functionalities of gels. At the same time, our study demonstrates potential industrial application prospect in removing pollutants efficiently (such as aromatic solvents and toxic dye removal).

**Electronic supplementary material:**

The online version of this article (10.1186/s11671-019-2865-6) contains supplementary material, which is available to authorized users.

## Background

The multicomponent supramolecular gel systems, as intriguing soft materials formed via H-bonding, donor–acceptor, metal ion coordination, and acid–base interactions, provide a flexible method to realize the functionalities of gels [1–3]. They have attracted more and more attention owing to their potential applications in the fields such as pollutant collection [4–6], oil spill treatment [5, 7], and advanced materials.

**Scheme 1:**
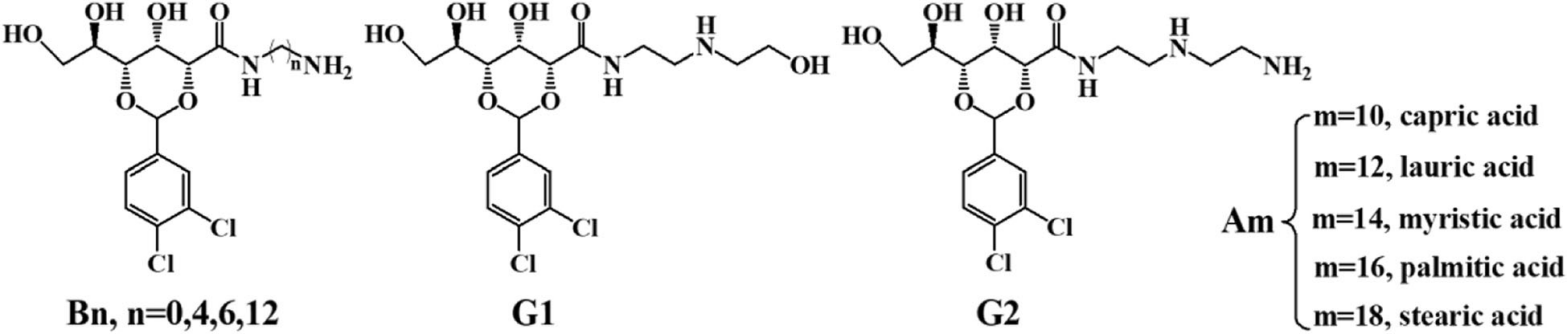
Structure of Bn, Gn, and aliphatic acid

Nowadays, much attention has been paid to environmental problems raised from the rapid industrial development [5, 8, 9]. When the hazardous waste such as industrial waste water, toxic dyes, and petroleum products are discharged as untreated effluents into rivers, lakes, and oceans, these pollutions threaten the fresh water ecology, affect aquatic life, and pollute the local sources of drinking water. For instance, the 2005 Jilin chemical plant explosions in Jilin City caused a large discharge of nitrobenzene into the Songhua River, which polluted the local sources of drinking water and the environment (https://en.wikipedia.org/wiki/2005_Jilin_chemical_plant_explosions). How to remove the toxic organic liquids which are heavier than water (e.g., nitrobenzene, o-dichlorobenzene) from their biphasic mixtures with industrial waste water easily and efficiently is a great challenge. At present, the possible materials for the treatment of such organic liquid pollutants involve adsorbents, chemical dispersants, polymeric solidifiers, and engineering bacteria [10–18]. However, all materials have some limitations in practice. For example, adsorbents are very efficient for the organic pollutants due to their high surface area, but the post-treatment is very expensive. Polymeric solidifiers cannot be mixed easily with organic pollutants, and the recovery of organic liquid from polymer gels is troublesome. Nowadays, the study of phase-selective gelation towards toxic organic liquids has become a hot topic [19–22]. For example, the first example of phase-selective gelator (PSG) was found by Bhattacharya and Krishnan-Ghosh with amino acid amphiphiles [23]. Vemula et al. Synthesized PSGS based on open-chain sugars [24]. Zeng et al. declared that leucine derivatives could gel crude oils in the presence of seawater at room temperature and a conceptually novel polar solvent-assisted approach to substantially boost the gelling capacities of different types of organogelators without resorting to any structural alteration [25, 26]. Our group previously reported the multifunctional gel systems B6-A18 based on the amine-acid two-component systems which showed room-temperature-phase-selective gelation and could simultaneously gel aromatic solvents (Scheme 1) [27, 28]. Furthermore, a majority of phase-selective gelators necessitated a heating–cooling process or a toxic co-solvent to obtain solutions before use. It was reported that an effective and ideal phase-selective gelator for practical applications must (1) be synthesized easily and at a low cost; (2) selectively, feasibly, and efficiently gel the organic phase in the presence of water at room temperature; (3) be environment-friendly; (4) be easily recovered from the gel involving the organic liquid pollutants; and (5) be recyclable and reusable [29]. So far, research on room-temperature-phase-selective gelation of the toxic organic liquids from their biphasic mixtures with water such as nitrobenzene is still limited [29].

Dyes are useful in the textile industry (e.g., paints, printing, drugs, and cosmetics). Most dyes are non-biodegradable and even at low concentrations, dyes still threaten the environment and the ecosystem [30–34]. Eliminating toxic dyes from the polluted water is therefore an important goal.

We recently reported the amine-acid two-component gelators Bn-Am (Scheme 1), which exhibited excellent gelation abilities towards certain organic solvents. Herein, based on these previous works [27, 28, 35], a few novel D-gluconic acetal-based derivatives Gn with a free amino or hydroxy group at the terminal position of a long alkyl chain and amino group in the middle of a long carbon chain were designed and synthesized. The amino or hydroxy group provides sites for H-bond interaction with aliphatic acids and for the formation of two-component gelators (Scheme 1).

The gelation properties and the gel performance could be flexibly tuned by changing the chain length of the alkyl group in aliphatic acid. Surprisingly, compared with Bn-Am, the two-component gel system Gn-Am could gel not only some organic solvents but also water at room temperature. Moreover, as shown in Additional file 1: Table S5, Bn-Am could gel organic solvents which have smaller densities than water (e.g., toluene, o-xylene, 1, 3, 5-trimethylbenzene and ethylbenzene) and Gn-Am showed strong gelation properties towards organic solvents which are heavier than water (e.g., nitrobenzene, o-dichlorobenzene, dichloromethane). Additionally, Gn-Am showed strong abilities to selectively gel nitrobenzene/o-dichlorobenzene from the mixture with wastewater (containing 0.5 M sodium nitrate and 0.5 M sodium sulfate) without employing co-solvents and a heating-cooling process. Moreover, the gel system Gn-Am at room temperature displayed high-efficiency self-healing properties. To the best of our knowledge, it was the very few examples of two-component gelators reported to be excellent room-temperature PSGs that could gel organic solvents and water. It was also the first example of a two-component gel system reported as excellent room-temperature PSGs used to remove nitrobenzene/o-dichlorobenzene from the wastewater directly in a powder form. Furthermore, other excellent functions of two-component gels including dye removal are also demonstrated. These findings provide a simple method for the design of multifunctional supramolecular gelators via an effective two-component gel strategy.

## Methods/Experimental

### Materials

D-Gluconic acid, 3, 4-dichlorobenzaldehyde, and β-hydroxyethylenediamine were purchased from Shanghai Jingchen Scientific Co., Ltd. The chemical reagents were commercially available and directly utilized without further purification. 2, 4-(3, 4-Dichloro) benzylidene methyl-d-gluconate was synthesized by the methods reported previously. [27]. Characterizations of a new compound *Gn* are provided in Additional file 1. Synthetic routes of *Gn* are shown in Additional file 1: Scheme S1. The detailed synthetic procedures and characterization data of *Gn* are given in Additional file 1.

### Preparation of the Two-Component Hydrogels

*Gn* (5 mmol) and aliphatic acid (5 mmol) were simultaneously added to 10 mL methanol. The resultant mixture was subsequently heated to reflux for 10 min leading to a clear solution and finally a white or faint yellow solid via vacuum evaporation. A certain amount of the two-component gelator was weighed in a test tube. The corresponding solvents was subsequently added, which was shook for 1 min and then the test tube stood for 8 h. Finally, the test tube was inversed to observe whether the solution inside could still flow [36]. Gelation was considered to have occurred when a homogeneous substance was obtained which exhibited no gravitational flow, and it was denoted by “G”. Solution and solid-like gel may coexist within a system as “partial gels (PG)”. Systems, in which only solution was obtained, were referred to as solution (S). In an insoluble system (I), gelators could not be dissolved. The critical gelation concentrations (CGCs) mean the minimum amount of gelators required to immobilize 1 mL of solvent.

### Field-emission scanning electron microscope (FESEM)

The morphologies of the xerogels were obtained by a Hitachi S-4800 SEM instrument operating at 3–5 kV. Samples were prepared by dropping the diluted solution of gels on the thin aluminum sheets and then dried under vacuum for 24 h. We coated the samples with a thin layer of Au before the experiment.

### FT-IR

IR spectra were collected by a FTS3000 spectrometer with KBr pellets. The xerogels were prepared by drying chlorobenzene gels on glass slides under vacuum for 24 h.

### Powder X-Ray Diffraction (PXRD)

PXRD diagrams of xerogels which were prepared from hydrogels were obtained by using a Bruker D8-S4 (CuKα radiation, *λ* = 1.546 Å). The *d* spacing values were calculated by Bragg’s law (*nλ* = 2*d* sinθ).

### Rheology Measurements

Rheology experiments were carried out with a strain-controlled rheometer (Anton Paar Physica MCR 301) equipped with steel-coated parallel-plate geometry (15 mm diameter). The gap distance was fixed at 0.5 mm. A solvent trapping device was placed above the plate and measurement was set at 20 °C in order to avoid solvent evaporation. The frequency sweep at a constant strain of 0.1% was obtained from 0.1 to 100 rad s^− 1^. Strain sweep was performed in the 0.01–1000% range at a constant frequency (1 Hz). The time sweep was conducted to observe the recovery property of the gel. First, a constant strain of 0.1% was applied on the sample. Then a constant strain of 100% was applied to destroy the sample. And then a constant strain (0.1%) was applied again. The storage modulus *G*’ and the loss modulus *G*” of the sample were monitored as functions of time in this experiment.

### Dye Removal Experiments

Five milligrams of the xerogels (*G1-Am*) prepared from the hydrogels by freeze drying was immersed in a single dye solution (5 mL, 0.1 mM) for 24 h containing the anionic dyes (e.g., acid fuchsin (AF), eosin Y (EY), methyl orange (MO)) or cationic dyes (e.g., malachite green (MG), methylene blue (MB), Rhodamine (RB)). The resultant mixture was subsequently centrifuged, and the concentration of dyes in the supernatant was monitored by UV–vis spectroscopy. The removal rate was calculated as (*C*_0_ − *C*_e_)/*C*_0_, where *C*_0_ (mg L^− 1^) was the initial concentration of dye in the solution and *C*_e_ (mg L^− 1^) was the equilibrium concentration. The maximum amount of dyes adsorbed at equilibrium *q*_e_ (mg g^− 1^) was calculated as *q*_e_ = (*C*_0_ − *C*_e_) × *V*/*W*, where *V* (L) was the solution volume and *W* (g) was the mass of xerogels. The changes in the dye concentration were also monitored by UV–vis spectroscopy. The adsorption isotherm was determined by immersing 5 mg of the xerogel into a MO or CV solution with varying concentrations for a week, and subsequently calculating the equilibrium adsorption capacity and concentration.

## Results and Discussion

### The Gelation Abilities of the Two-Component Gel System at Room Temperature

**Fig. 1:**
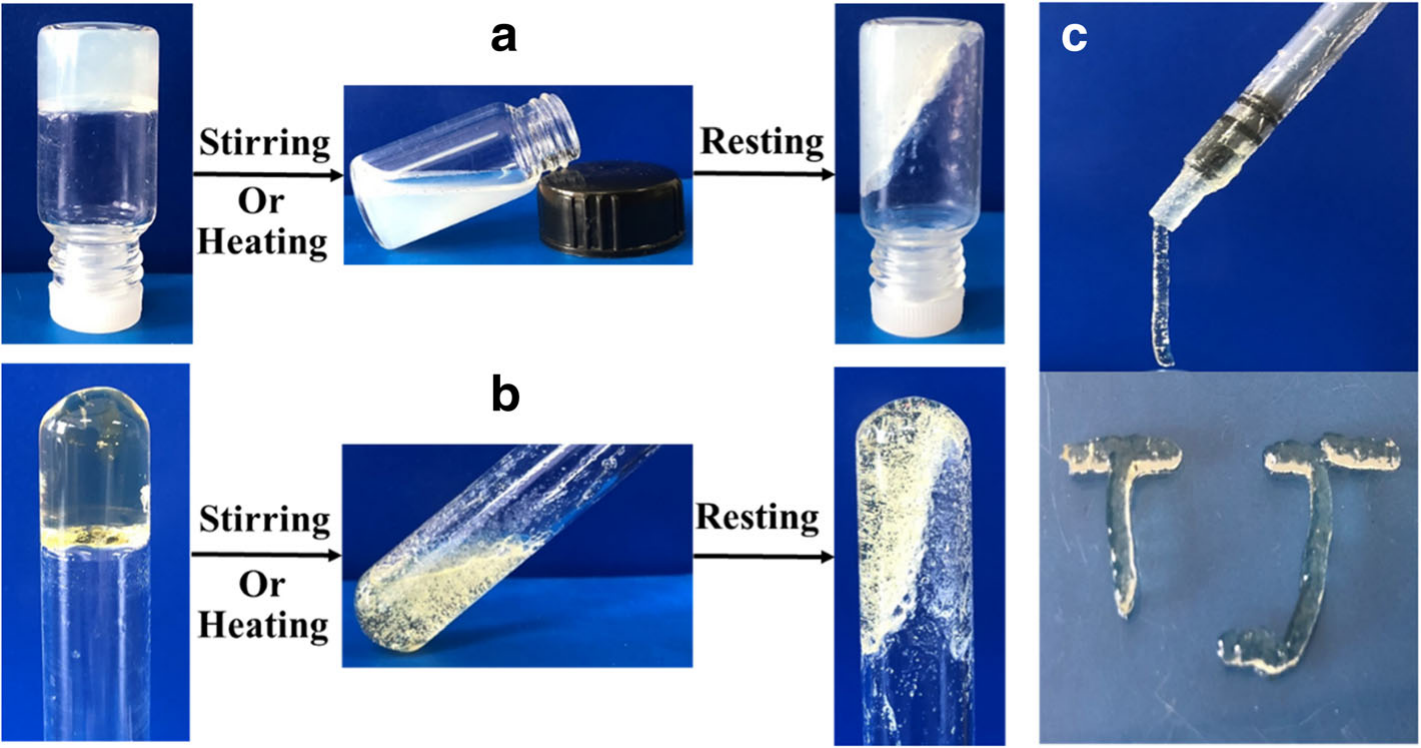
**a** Reversible sol-to-gel transitions of *G1-A16* room-temperature hydrogel stimulated by shaking or heating at 20 mg mL^− 1^. **b** Reversible sol-to-gel transitions of *G1-A16* room-temperature nitrobenzene gel stimulated by shaking or heating at 20 mg mL^− 1^. **c** Extrusion of *G1-A16* room-temperature nitrobenzene gel from a syringe

Initially, the gelation tests for Gn-Am in some solvents were summarized in Additional file 1: Table S1-S4. All the gels reported here were generated by using a 1:1 or 1:2 (the molar concentration of the two-component gel) ratio of Gn/aliphatic acids as the two-component gelators. The gelation abilities of the two-component gelators varied depending upon the structure of the single component. In particular, the gelation abilities of the Gn-Am at room temperature were greatly enhanced compared with that of a single Gn. It was found that G1-Am and G2-A16 were efficient room-temperature gelators in certain solvents such as nitrobenzene, o-dichlorobenzene, dichloromethane, toluene, and water, while G1 and G2 cannot gelate any testing solvent at room temperature. Except for G2-A16, G2-Am did not exhibit room-temperature gelation abilities in all the tested solvents (Additional file 1: Table S2 and S4). Intriguingly, the G1-Am gels exhibited high-performance self-healing properties. All the G1-Am room-temperature gels can undergo an instant self-healing process (within 5 s) upon mechanical damage. The self-healing properties of G1-A16 gels were further demonstrated by shaking/resting tests at room temperature. For instance, the G1-A16 hydrogel and nitrobenzene gel (2.0%, *w*/*v*) were shaken vigorously until a homogeneous solution was resulted, and then the solutions were rested at room temperature, leading to re-formation of gels spontaneously within 1 min (Fig. 1a, b). A similar behavior was also observed for gel formation with G1-A16 in o-dichlorobenzene. Meanwhile, G1-A16 can gelate nitrobenzene/o-dichlorobenzene at room temperature at a concentration as low as 0.45 wt% and 0.72 wt% respectively. These findings established the basis for the real-life applications of these gelators in the phase-selective gelation of nitrobenzene/o-dichlorobenzene from wastewater at room temperature. Also, G1-A16/nitrobenzene gels showed highly transparent and excellent viscoelasticity that allow it to be easily processed such as extrusion from a syringe (Fig. 1c). The high transparency, enhanced viscoelasticity, and the rapid self-healing properties of these gels make them attractive for developing flexible optical devices [37]. Furthermore, it was shown that G1-A16 xerogels obtained from nitrobenzene/water could adsorb toxic dyes from the polluted water, indicating a promising application in the field of water purification. However, the two-component gel systems obtained by mixing aliphatic acids with G2 except for A16 (palmitic acid), did not show enhanced room-temperature gelation abilities and interesting properties mentioned above. It indicated that the terminal hydroxyl attached to the side alkyl chain in Gn was critical to tune the properties of a two-component gel system.

### Rheology of Self-Sealing Two-Component Gel System

**Fig. 2:**
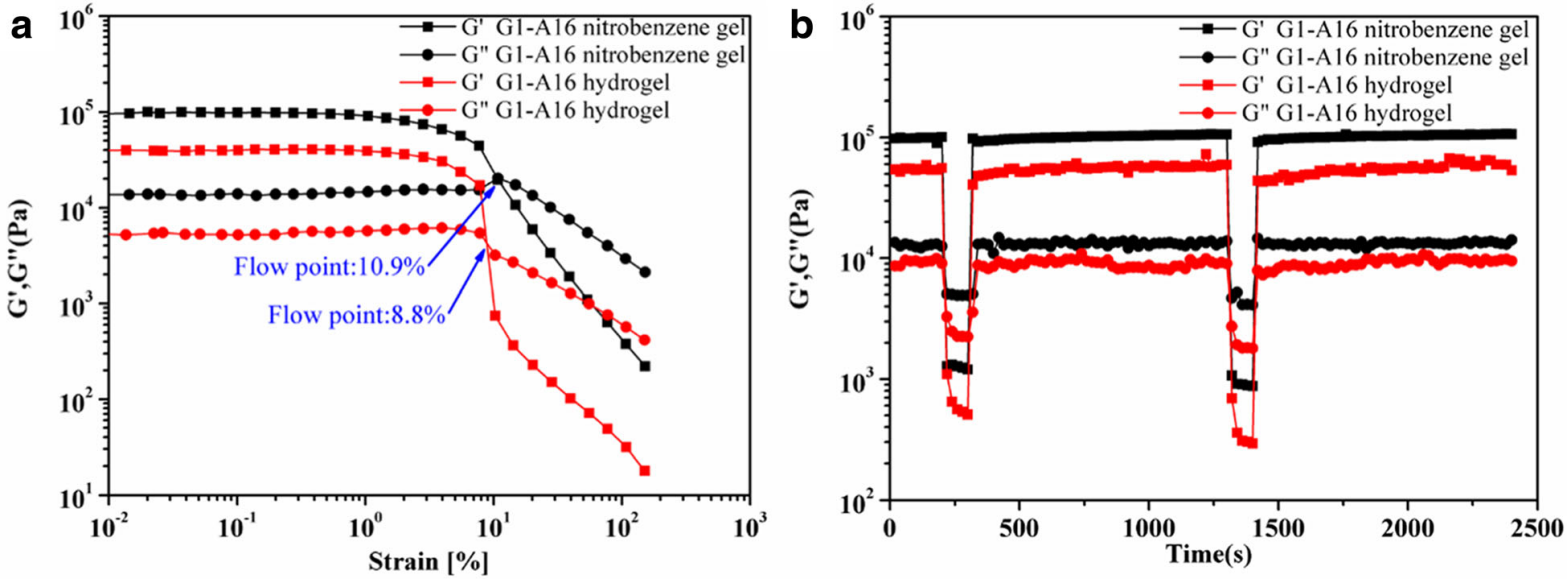
**a** Rheological strain sweep was performed from 0.01 to 100% with 1 Hz at 20 °C (*G1-A16*: 2% *w*/*v*). **b** Time scan tests were performed with an alternating strain of 0.1 and 100% with 1 Hz at 20 °C (*G1-A16*: 2% *w*/*v*)

Mechanical properties of the two-component gel systems at room temperature were studied by oscillatory rheological measurements. For all the gel samples, the storage modulus *G*’ has a larger magnitude than the loss modulus *G*” in the overall range of frequency sweep and the linear viscoelastic region (LVR) of the strain sweep, confirming their gel nature (Additional file 1: Figure S1) [38]. The strain sweep showed that G1-A16/nitrobenzene gels could tolerate a much larger strain (flowing point = 10.9%) than the G1-A16/hydrogel (flowing point = 8.8%) (Fig. 2). The recovery properties of the gels were examined by time scan tests under alternating strain of 0.1% and 100% (Fig. 2 and Additional file 1: Figure S1). Obviously, G1-Am gels exhibited excellent self-healing abilities, and the *G*’ values could recover to their original values immediately after the cessation of the destructive strain. The damage recovery process could be repeated for at least two cycles without any reduction in the value of *G*’ and *G*”. Almost all G1-Am gels could repair themselves within 5 s. As shown in the Additional file 1: Figure S1 (g), the *G*’ values of G1-A10, G1-A12, G1-A16, and G1-A18/nitrobenzene gels were 1460, 5300, 100,000, and 107,000 Pa. These results revealed the mechanical properties of two-component gel systems at room temperature can be regulated via the finely tuning of the alkyl chain length of Am.

### Room-Temperature-Phase-Selective Gelation in the Removal of Nitrobenzene

**Fig. 3:**
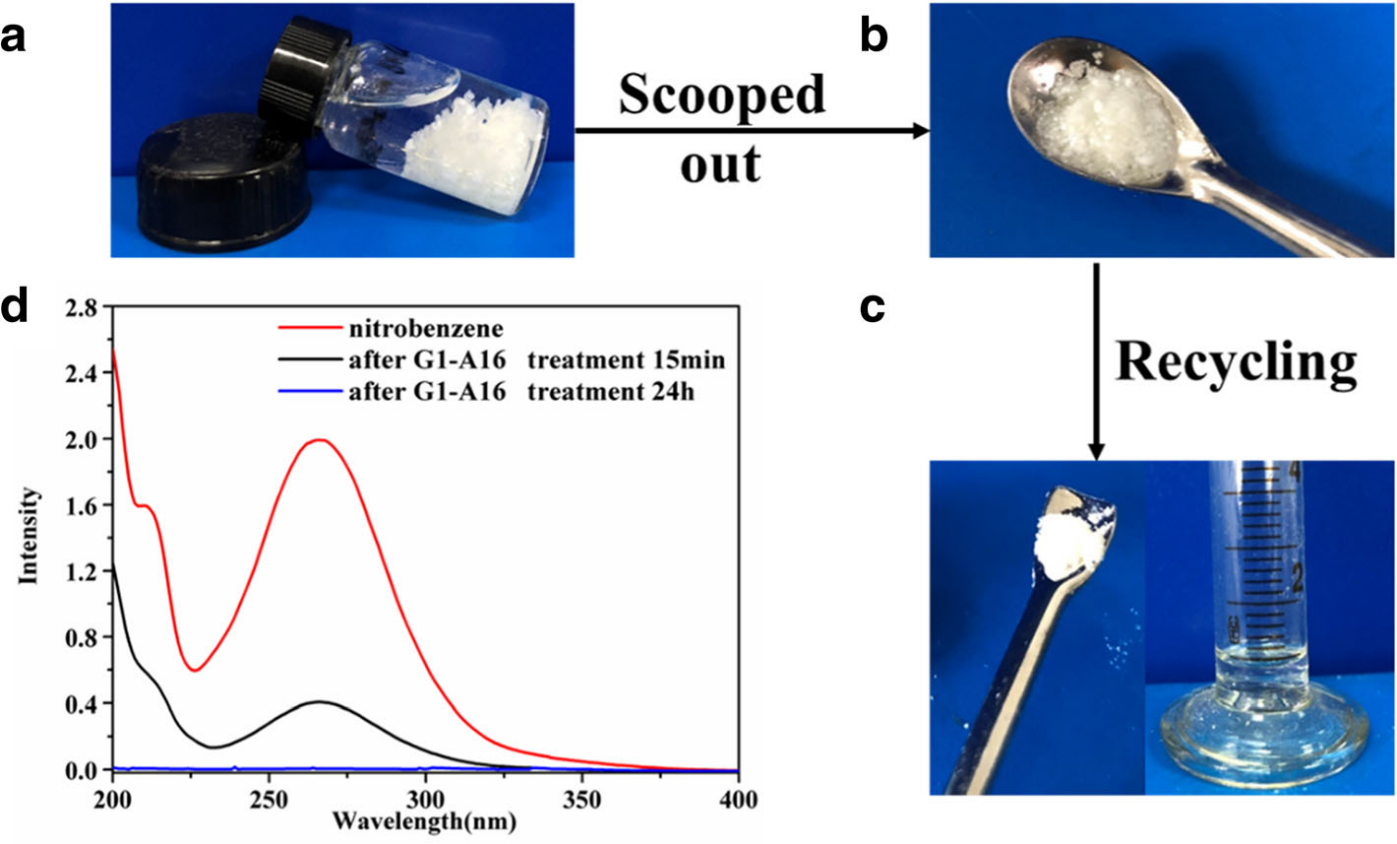
**a** Specific gelation of the nitrobenzene phase using *G1-A16* (*G1-A16* is 40 mg) as a phase selection gel in a two-phase mixture of nitrobenzene and wastewater (1 mL/3.0 mL, NaNO_3_ and Na_2_SO_4_ concentration of wastewater is 0.5 M respectively) by mechanical shaking. **b** Separation of the formed gel–water mixture into the nitrobenzene gel via simple scooping out. **c** Recovery of nitrobenzene from the *G1-A16* nitrobenzene gel via distillation, and purification of the restored gelator by recrystallization. **d** UV–vis spectra of the nitrobenzene before and after removal over the *G1-A16*

For the *G1-A16* gel system at room temperature reported in this work, via simple mechanical shaking, the two-component gelators were fully dispersed in the nitrobenzene phase, which further facilitated complete gel formation in wastewater (containing 0.5 M sodium nitrate and 0.5 M sodium sulfate). For example, as shown in Fig. 3, 40 mg *G1-A16* powders were directly added to a mixture of nitrobenzene/water (1 mL/3 mL containing 0.5 M sodium nitrate and 0.5 M sodium sulfate) in a glass vial. Further vigorous shaking was carried out to facilitate the sufficient dissolution of *G1-A16* powders in nitrobenzene. As shown in Fig. 3a, at room temperature, the resulting feculent mixture was rested for 15 min leading to a gel-like chunk (Fig. 3a). The gel-like chunk was scooped out easily by a spoon (Fig. 3b). Furthermore, the two-component gelators could be recycled via a simple distillation process and the nitrobenzene solvents recovered (the average recovery ratio was 85%). The restored two-component gelators could be purified by recrystallization (Fig. 3c). As shown in Fig. 3d, the treated water by *G1-A16* only contained trace amounts of nitrobenzene. Similarly, Additional file 1: Figure S3 shows that *G1-A16* could be used to remove o-dichlorobenzene. To the best of our knowledge, this is the first example of the two-component gelators that had the potential application in the real-life recovery of nitrobenzene/o-dichlorobenzene.

#### Removal of Dyes

**Fig. 4:**
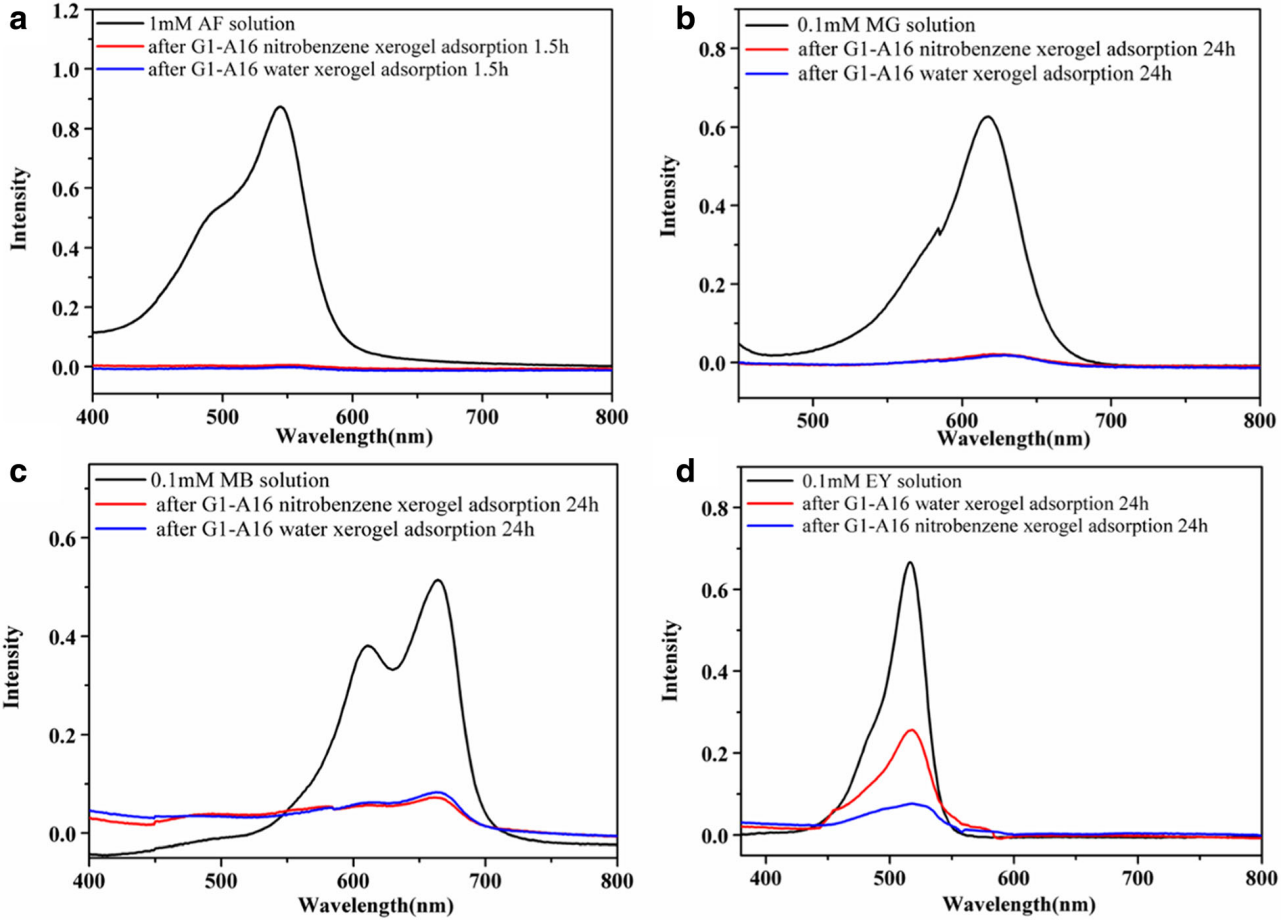
UV–vis spectra of the dye solutions before and after removal over the *G1-A16* xerogels: **a** AF, **b** MG, **c** MB, and **d** EY

The industrial wastewater containing dye molecules are classical refractory organic pollutants. These effluents cause serious pollution to the environment, and the treatment of such pollutants has also been a field of focused research recently. In our case, *G1-A16* xerogels obtained from nitrobenzene or water were further explored for the removal of toxic dyes (anionic dyes: acid fuchsin (AF), eosin Y (EY), methyl orange (MO); cationic dyes: malachite green (MG), methylene blue (MB), rhodamine (RB), structures shown in Additional file 1: Figure S3) from water in detail. Interestingly, the xerogels showed excellent adsorption capacity for all testing dyes except for MO (Fig. 4 and Additional file 1: Figure S4). For instance, 5 mg *G1-A16* xerogel obtained from nitrobenzene were poured into the aqueous solution of MG (5 mL, the concentration at 0.1 mmol/L) at room temperature. The adsorption behavior of the xerogel was monitored by UV–vis spectroscopy measurement of the aqueous solution. In Fig. 4b, after 24 h, about 99.73% of MG were adsorbed. It was shown in Fig. 4a that *G1-A16* xerogel was the efficient sorbents for AF (the concentration is 1 mM). AF was completely absorbed only 1.5 h. In addition, about 86.02% MB was absorbed by *G1-A16* xerogels even after 24 h (Fig. 4c). Furthermore, the maximum adsorption capacity of the two types of xerogels was examined (Additional file 1: Table S6). Encouragingly, the maximum amount of AF adsorbed for the *G1-A16*/nitrobenzene and *G1-A16*/water xerogels reached 610.75 and 594.09 mg g^−1,^ respectively (Additional file 1: Table S6). This large dye-removal amount is very rare for supramolecular gel systems [5]. Figure 4d and Additional file 1: Figure S4 and Table S6 indicate that the adsorption capacity of the *G1-A16* nitrobenzene xerogels was stronger than that of the *G1-A16* xerogels obtained from hydrogel for some toxic dye (e.g., EY, RB). Interestingly, the anionic dye-adsorption abilities of *G1-A16* xerogels were stronger than those of *B6-A16* xerogels. In contrast, *B6-A16* xerogels showed strong adsorption properties of the cationic dyes (Additional file 1: Table S6).

### FT-IR

**Fig. 5:**
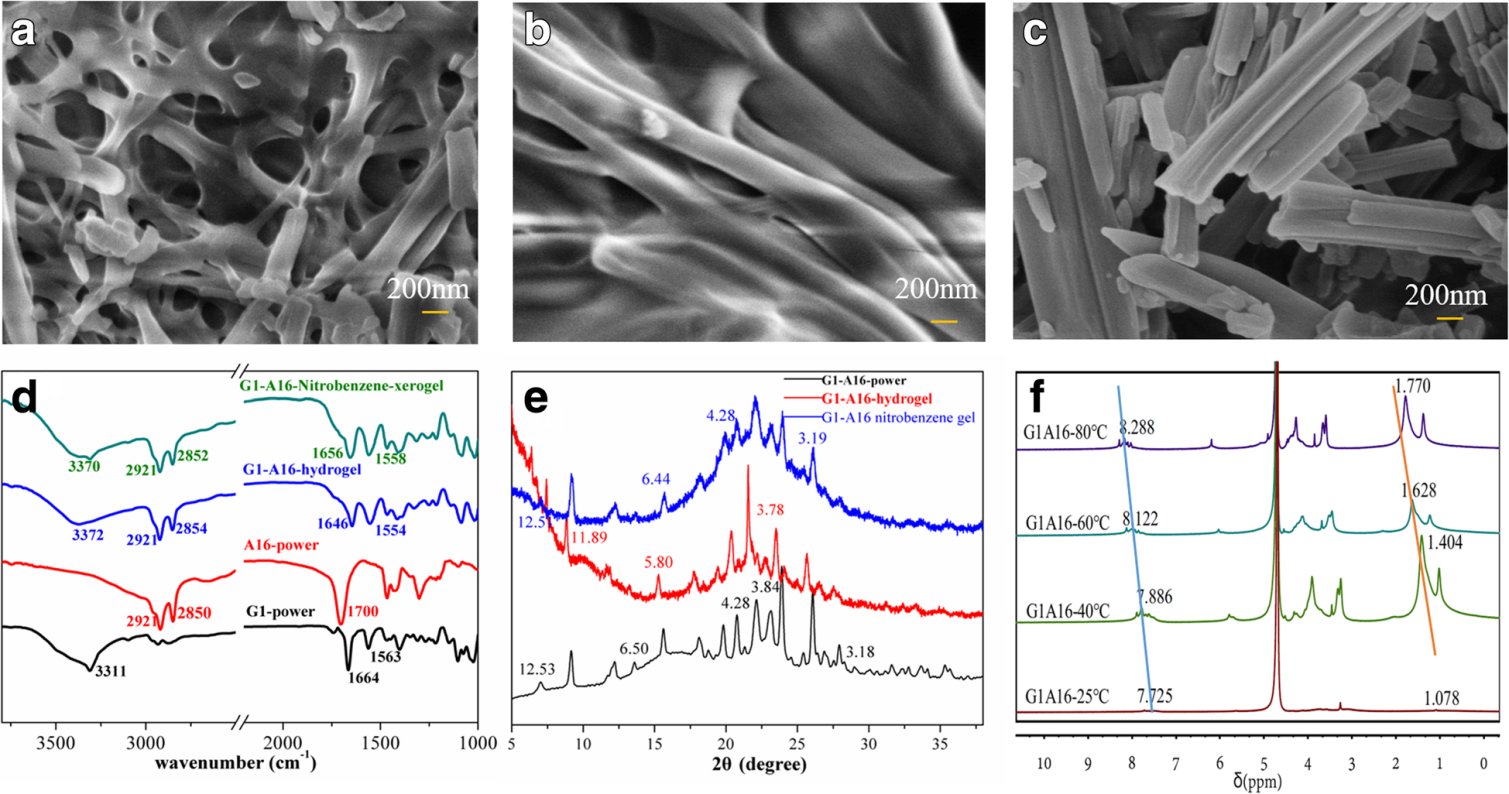
**a** SEM image of xerogels prepared by *G1-A16* nitrobenzene gel. **b** SEM image of xerogels prepared by *G1-A16* hydrogel. **c** SEM image of *G1* power. **d** FT-IR spectra of *G1-A16* from chlorobenzene xerogel, hydrogel (2.0% *w*/*v*), *A16* power and *G1* power. **e** X-ray diffraction pattern of the xerogel of *G1-A16* from chlorobenzene xerogel, hydrogel (2.0% *w*/*v*). **f** *G1-A16* power, ^1^H NMR spectra of *G1-A16* (2.0% *w*/*v*) in D_2_O at different temperatures

To reveal the mechanism of on the self-assembly of the *G1-Am* gel system, the FT-IR investigations of *G1* powder, *A16* powder, and *G1-A16* xerogels were performed (Fig. 5d). In the FT-IR spectra, the absorption bands of the asymmetric and symmetric CH_2_ stretching vibrations of the side alkyl chains of the *G1-A16*/nitrobenzene and *G1-A16*/water xerogels were observed at 2921 cm^− 1^, 2852 cm^− 1^ and 2921 cm^− 1^, 2854 cm^− 1^, respectively, suggesting that the alkyl chains are all in the trans form and there are strong van der Waals forces between the components [39, 40]. In the FT-IR spectra of *G1-A16*/nitrobenzene and water xerogels, the bands of OH (NH) appeared at 3370 cm^− 1^ and 3372 cm^− 1^ respectively, which were observed at 3311 cm^− 1^ for the G1 power. Furthermore, for *G1-A16*/nitrobenzene and water xerogels, the band of the carbonyl group of the stearic acid component at 1700 cm^− 1^ disappeared, indicating that all carbonyls participated in H-bonding interaction in the two-component gel system [41–43]. The amide I and II bands of the xerogels were found at 1656 cm^− 1^, 1558 cm^− 1^ and 1646 cm^− 1^, 1554 cm^− 1^ respectively, also implying the formation of the intermolecular hydrogen bonding [43–45].

### Powder X-Ray Diffraction (PXRD)

The XRD patterns of *G1-A16* nitrobenzene xerogel showed *d* spacing values of 1.25, 0.64, 0.43, and 0.32 nm (Fig. 5e) in a ratio of 1:1/2:1/3:1/4, indicating the lamellar arrangements [46, 47]. Similarly, the XRD patterns of *G1-A16*/water xerogel and *G1-A16* powder suggested the lamellar arrangements. Additionally, the *d* values of 0.38 nm was the characteristic of π–π stacking force of the benzene rings [48–50]. Moreover, the *d* value of 0.43 nm was ascribed to the packing of the alkyl chains [51, 52]. It reveals that the driving forces for the self-assembly involve the π–π stacking force of the benzene rings and van der Waals force of the alkyl chains in the solution system.

### ^1^H NMR

In order to gain further understanding of the possible driving force in the self-assembly of *G1-A16* for gel formation, temperature-dependent ^1^H NMR spectroscopic studies were also performed in D_2_O-d6. As shown in Fig. 5f, ^1^H NMR spectra of the *G1-A16* (2.0% *w*/*v*) in D_2_O at different temperatures were compared. One of the H-shifts on the benzene ring of *G1-A16* hydrogel appeared at 8.288 ppm in pure D_2_O at 80 °C. The aromatic proton signals showed an up-field shift (blue line in Fig. 5f) when the temperature decreased, which provided the support for the existence of π–π interactions between the phenyl groups of *G1-A16* in the gel state. Concurrently, the ^1^H NMR spectral signals of the alkyl chain protons were observed to shift up-field when the temperature reduced (orange line in Fig. 5f). Accordingly, these results reveal that the main driving force for the self-assembly of *G1-A16* in water is the combined interactions of π–π and van der Waals.

These results suggested that the excellent properties of the two-component *G1-Am* gels originated from their highly ordered structures which formed based on the synergistic effect of intermolecular hydrogen bonding, van der Waals, and π–π stacking.

## Conclusions

In summary, we have designed a novel multifunctional two-component gel system, which exhibited highly efficient self-healing and room temperature-phase-selective properties, and potential applications in the fields of waste water treatment. The viscoelasticity and self-healing properties of gels can be successfully tuned by changing the length of the alkyl chain of the aliphatic acid component. Surprisingly, the *G1-Am* gel system could gel four organic solvents and water at room temperature. Moreover, the powders of the *G1-A16* could directly gel nitrobenzene/o-dichlorobenzene from their biphasic mixtures with wastewater at room temperature via simple mechanical shaking. In addition, the xerogels obtained from *G1-A16* gel can be used to effectively remove toxic dyes (anionic dyes: AF, EY; cationic dyes: MG, MB, RB) from their aqueous solutions. Further studies on the relationship of gel properties and the component structure and exploring applications of these materials are still in progress.

## Additional file

Additional file 1: **Scheme S1.** The synthetic routes of *Gn*. **Table S1.** Gelation behavior of gelators *G1-Am* (the molar ratio is 1:1) in various solvents at room temperature (about 25 °C). **Table S2.** Gelation behavior of gelators *G2-Am* (the molar ratio is 1:1) in various solvents at room temperature (about 25 °C). **Table S3.** Gelation behavior of gelators *G1-Am* (the molar ratio is 1:2) in various solvents at room temperature (about 25 °C). **Table S4.** Gelation behavior of gelators *G2-Am* (the molar ratio is 1:2) in various solvents at room temperature (about 25 °C). **Figure S1.** Oscillatory rheological study of gel from *G1-Am* (the molar ratio is 1:2, 2%, *w*/*v*): (a) *G1-A12* nitrobenzene gel, (b) *G1-A14* nitrobenzene gel, (c) *G1-A16* nitrobenzene gel (d) *G1-A18* nitrobenzene gel (e) *G1-A16* hydrogel, (f) *G1-A16* hydrogel, (g) Frequency sweep of nitrobenzene gel from *G1-Am* with a fixed strain (0.1%) at 20 °C. **Figure S2.** (a) Specific gelation of the o-dichlorobenzene phase using *G1-A16* (*G1-A16* is 40 mg mL-1) as a phase selection gel in a two-phase mixture of o-dichlorobenzene and wastewater (1 mL/3.0 mL, NaNO_3_ and Na_2_SO_4_ concentration of wastewater is 0.5 M) by mechanical shaking. (b) Separation of the formed gel–water mixture into the o-dichlorobenzene gel via simple scooped out. (c) Recovery of o-dichlorobenzene from the *G1-A16* nitrobenzene gel via distillation, and purification of the restored gelator by recrystallization. **Figure S3.** The molecular structures of dyes. **Figure S4.** Time-dependent UV–vis spectroscopy measurement of the *G1-A16* xerogels-treated *RB* aqueous solution (a), *MO* (b), *AF* (c). **Table S5.** Maximum adsorption capacity of xerogels. (DOCX 1791 kb)

## Abbreviations

^1^H NMR: H nuclear magnetic resonance
AF: Acid fuchsin
EY: Eosin Y
FESEM: Field-emission scanning electron microscope
FT-IR: Fourier transform infrared spectroscopy
MB: Methylene blue
MG: Malachite green
MO: Methyl orange
PXRD: Powder X-ray diffraction
RB: Rhodamine
