# Supplementary material for: Smart Materials for Environmental Remediation Based on Two-Component Gels: Room-Temperature-Phase-Selective Gelation for the Removal of Organic Pollutants Including Nitrobenzene/O-Dichlorobenzene, and Dye Molecules from the Wastewater
Source: Nanoscale Res Lett. 2019 Feb 1;14:42. doi: 10.1186/s11671-019-2865-6 (PMC6358627; doi:10.1186/s11671-019-2865-6)
Supplement: Supplementary file 1 — Scheme S1. The synthetic routes of Gn. Table S1. Gelation behavior of gelators G1-Am (the molar ratio is 1:1) in various solvents at room temperature (about 25 °C). Table S2. Gelation behavior of gelators G2-Am (the molar ratio is 1:1) in various solvents at room temperature (about 25 °C). Table S3. Gelation behavior of gelators G1-Am (the molar ratio is 1:2) in various solvents at room temperature (about 25 °C). Table S4. Gelation behavior of gelators G2-Am (the molar ratio is 1:2) in various solvents at room temperature (about 25 °C). Figure S1. Oscillatory rheological study of gel from G1-Am (the molar ratio is 1:2, 2%, w/v): (a) G1-A12 nitrobenzene gel, (b) G1-A14 nitrobenzene gel, (c) G1-A16 nitrobenzene gel (d) G1-A18 nitrobenzene gel (e) G1-A16 hydrogel, (f) G1-A16 hydrogel, (g) Frequency sweep of nitrobenzene gel from G1-Am with a fixed strain (0.1%) at 20 °C. Figure S2. (a) Specific gelation of the o-dichlorobenzene phase using G1-A16 (G1-A16 is 40 mg mL-1) as a phase selection gel in a two-phase mixture of o-dichlorobenzene and wastewater (1 mL/3.0 mL, NaNO3 and Na2SO4 concentration of wastewater is 0.5 M) by mechanical shaking. (b) Separation of the formed gel–water mixture into the o-dichlorobenzene gel via simple scooped out. (c) Recovery of o-dichlorobenzene from the G1-A16 nitrobenzene gel via distillation, and purification of the restored gelator by recrystallization. Figure S3. The molecular structures of dyes. Figure S4. Time-dependent UV–vis spectroscopy measurement of the G1-A16 xerogels-treated RB aqueous solution (a), MO (b), AF (c). Table S5. Maximum adsorption capacity of xerogels. (DOCX 1791 kb) [file 11671_2019_2865_MOESM1_ESM.docx]

**Supporting information**

**Smart materials for environmental remediation based on two-component gels: Room-temperature-phase-selective gelation in the removal of nitrobenzene/o-dichlorobenzene, and dye removal**

**Jing Zhang ^1,2,3^, Jiahui Liu^4^,** **Ciqing Tong^5^, Shipeng Chen^1,2^,** **Baohao Zhang**^1,2^, **Bao Zhang ^1,2*^ and Jian Song ^1,2*^**

^1^ School of Chemical Engineering and Technology, Tianjin University, Tianjin 300350, China; [zhangjing2014@tju.edu.cn](mailto:zhangjing2014@tju.edu.cn); 570305csp@sina.cn; [zhangbaohao@tju.edu.cn](mailto:zhangbaohao@tju.edu.cn); [baozhang@tju.edu.cn](mailto:baozhang@tju.edu.cn); [songjian@tju.edu.cn](mailto:songjian@tju.edu.cn)

^2^ The Co-Innovation Center of Chemistry and Chemical Engineering of Tianjin, Tianjin 300072, China

^3^ Renai College of Tianjin University, Tianjin 301636, China

^4^ Max Planck Institute for Polymer Research, Ackermannweg 10, 55128 Mainz, Germany; [liujiahui@mpip-mainz.mpg.de](mailto:liujiahui@mpip-mainz.mpg.de)

^5.^Department of Supramolecular and Biomaterials Chemistry, Leiden Institute of Chemistry, Leiden University, P.O. Box 9502, 2300 RA, Leiden, The Netherlands; [c.tong@lic.leidenuniv.nl](mailto:c.tong@lic.leidenuniv.nl)

*****Correspondence: baozhang@tju.edu.cn; songjian@tju.edu.cn.

**1. Experimental details**

**Synthetic routes of Gn:**

**Scheme S1**. The synthetic routes of Gn

**Synthetic details:**

The synthesis and characterization of the precursors 2, 4-(3, 4-dichloro) benzylidene Methy-D-Gluconate(**A**) as reported previously.[1]

5 g (0.014 mol) 2, 4-(3, 4-dichloro) benzylidene Methy-D-Gluconate was dissolved in 50 mL methanol, then 2.19 g (0.021 mol) 1-aminohexane and 0.01 g DMAP (0.008 mmol) were added. The reaction mixture was stirred for 12h and then 20 mL water was added. Subsequently，the white solid was collected by filtration. The filter cake was washed with water for twice and recrystallized with methanol to obtain compound **G1** with a yield of 60%. Similarly, **G2** was obtained from 2,4-(3,4-dichloro) benzylidene Methy-D-Gluconate with β-hydroxyethylenediamine respectively and purified by the same method.

1.3 Chemical characterization

**G1**：^1^H NMR(400MHz，DMSO-d6)：δ7.87(s，1H，CO-NH)，7.70-7.65(d，1H，Ar-H)，7.57-7.53(d，1H，Ar-H)，7.45-7.51(t，1H，Ar-H)，5.68(s，1H，OCHO)，4.78-4.83(m，1H，NH)，4.75-4.71(d，1H，OH)，4.47-4.43(d，2H，OH)，4.37(s，1H，CH)，4.00(s，1H，OH)，3.77(d，1H，CH_2_)，3.65(s，1H，CH)，3.55(m，1H，CH)，3.45-3.41(d，1H，CH_2_),3.25-3.20(d，1H，CH_2_)，2.60-2.54(m，1H，CH_2_).

**G2**：^1^H NMR(400MHz，DMSO-d6)：δ7.86(s，1H，CO-NH)，7. 68-7.66(d，1H，Ar-H)，7.55-7.52(d，1H，Ar-H)，7.48-7.45(t，1H，Ar-H)，5.68(s，1H，OCHO)，4.37-4.37(d，1H，OH)， 3.99(s，1H，CH)， 3.78-3.75(d，1H，CH_2_)，3.67(s，1H，CH)，3.57(m，1H，CH)，3.57-3.56(d，1H，CH_2_)，3.54(d，1H，CH_2_)，3.44-3.40(m，1H，CH2)，3.26(m，1H，NH)，3.15(m，1H，NH_2_).

**2. Additional data**

**Table S1** Gelation behavior of gelators **G1-Am** (the molar ratio is 1:1) in various solvents

at room temperature (about 25℃)

| solvent | **G1** | **G1-A8** | **G1-A10** | **G1-A12** | **G1-A14** | **G1-A16** | **G1-A18** |
| --- | --- | --- | --- | --- | --- | --- | --- |
| water | I | I | PG | OG[31.7℃] [0.86%] | I | OG[37.4℃] [0.54%] | OG[38.8℃] [0.56%] |
| nitrobenzene | I | S | TG[52.9℃] [0.65%] | TG[56.9℃] [0.81%] | PG | TG[51.4℃] [0.45%] | TG[40.4℃] [0.83%] |
| [o-dichlorobenzene](file:///C:\\Users\\%E5%BC%A0%E9%9D%99\\AppData\\Local\\youdao\\dict\\Application\\7.5.2.0\\resultui\\dict\\?keyword=o-dichlorobenzene) | I | S | PG | OG[66.2℃] [1.30%] | PG | OG[50.1℃] [0.72%] | PG |
| toluene | I | I | PG | PG | I | TG[51.6℃] [3.2%] | PG |
| o-xylene | I | I | PG | PG | PG | PG | PG |
| chlorobenzene | I | I | PG | PG | I | PG | PG |
| dichloromethane | I | I | OG[70.1℃] [0.63%] | OG[59.8℃] [0.67%] | OG[50.4℃] [0.83%] | OG[55.4℃] [0.67%] | PG |
| methanol | I | I | I | I | I | I | I |
| acetone | I | I | I | I | I | I | I |
| acetonitrile | I | I | I | I | I | I | I |
| THF | I | I | I | I | I | I | I |

Gel Concentration: 2.5 % (w/v). OG: opaque gel; TG: Transparent gel; P: precipitate; S: solution. I: Insoluble. OG[Tgel] [CGCS].

**Table S2** Gelation behavior of gelators **G2-Am** (the molar ratio is 1:1) in various solvents

at room temperature (about 25℃)

| solvent | **G2** | **G2-A8** | **G2-A10** | **G2-A12** | **G2-A14** | **G2-A16** | **G2-A18** |
| --- | --- | --- | --- | --- | --- | --- | --- |
| water | I | I | I | I | I | I | I |
| nitrobenzene | I | I | I | I | I | I | I |
| [o-dichlorobenzene](file:///C:\Users\%E5%BC%A0%E9%9D%99\AppData\Local\youdao\dict\Application\7.5.2.0\resultui\dict\?keyword=o-dichlorobenzene) | I | I | I | PG | PG | OG[42.1℃] [2.61%] | PG |
| toluene | I | I | I | I | I | I | I |
| o-xylene | I | I | I | I | I | I | I |
| chlorobenzene | I | I | I | I | I | OG[48.3℃] [3.69%] | I |
| dichloromethane | I | I | I | I | I | I | I |
| methanol | I | I | I | I | I | I | I |
| acetone | I | I | I | I | I | I | I |
| acetonitrile | I | I | I | I | I | I | I |
| THF | I | I | I | I | I | I | I |

Gel Concentration: 2.5 % (w/v). OG: opaque gel; TG: Transparent gel; P: precipitate; S: solution. I: Insoluble. OG[Tgel] [CGCS].

**Table S3** Gelation behavior of gelators **G1-Am** (the molar ratio is 1:2) in various solvents

at room temperature (about 25℃)

| solvent | **G1** | **G1-A8** | **G1-A10** | **G1-A12** | **G1-A14** | **G1-A16** | **G1-A18** |
| --- | --- | --- | --- | --- | --- | --- | --- |
| water | I | I | I | I | OG[36.1℃] [1.3%] | OG[39.7℃] [1.3%] | PG |
| nitrobenzene | I | S | TG[52.9℃] [0.65%] | TG[56.9℃] [0.81%] | TG[88℃] [1.2%] | TG[74.3℃] [1.3%] | TG[52.8℃] [%] |
| [o-dichlorobenzene](file:///C:\Users\%E5%BC%A0%E9%9D%99\AppData\Local\youdao\dict\Application\7.5.2.0\resultui\dict\?keyword=o-dichlorobenzene) | I | S | I | I | TG[89.8℃] [1.7%] | TG[38.1℃] [1.3%] | TG[37.4℃] [%] |
| toluene | I | I | I | I | PG | TG[34.9℃] [%] | I |
| o-xylene | I | I | I | I | PG | PG | I |
| chlorobenzene | I | I | PG | PG | I | PG | PG |
| dichloromethane | I | I | TG[47.0℃] [0.95%] | TG[48.2℃] [1.1%] | TG[42℃] [0.87%] | OG[45℃] [0.77%] | OG[43.1℃] [1.25%] |
| methanol | I | I | I | S | S | I | I |
| acetone | I | I | I | I | I | I | I |
| acetonitrile | I | I | I | I | I | I | I |
| THF | I | I | I | I | I | I | I |

Gel Concentration: 2.5 % (w/v). OG: opaque gel; TG: Transparent gel; P: precipitate; S: solution. I: Insoluble. OG[Tgel] [CGCS].

**Table S4** Gelation behavior of gelators **G2-Am** (the molar ratio is 1:2) in various solvents

at room temperature (about 25℃)

| solvent | **G2** | **G2-A8** | **G2-A10** | **G2-A12** | **G2-A14** | **G2-A16** | **G2-A18** |
| --- | --- | --- | --- | --- | --- | --- | --- |
| water | I | I | I | I | I | I | I |
| nitrobenzene | I | I | I | I | I | I | I |
| [o-dichlorobenzene](file:///C:\Users\%E5%BC%A0%E9%9D%99\AppData\Local\youdao\dict\Application\7.5.2.0\resultui\dict\?keyword=o-dichlorobenzene) | I | I | I | I | I | I | I |
| toluene | I | I | I | I | I | I | I |
| o-xylene | I | I | I | I | I | I | I |
| chlorobenzene | I | I | OG[45.7℃] [1.25%] | OG[45℃] [ %] | I | I | I |
| dichloromethane | I | I | I | I | I | I | I |
| methanol | I | I | S | I | I | I | I |
| acetone | I | I | I | I | I | I | I |
| acetonitrile | I | I | I | I | I | I | I |
| THF | I | I | S | S | S | I | I |

Gel Concentration: 2.5 % (w/v). OG: opaque gel; TG: Transparent gel; P: precipitate; S: solution. I: Insoluble. OG[Tgel] [CGCS].

**Table S5** Gelation behavior of gelators **G1-Am**, **B6-Am** (the molar ratio is 1:1) in various solvents at room temperature (about 25℃)

| solvent | **G1-A12** | **G1-A16** | **G1-A18** | **B6-A12** | **B6-16** | **B6-A18** |
| --- | --- | --- | --- | --- | --- | --- |
| water | OG | OG | OG | I | I | I |
| nitrobenzene | TG | TG | TG | I | I | I |
| [o-dichlorobenzene](file:///C:\Users\%E5%BC%A0%E9%9D%99\AppData\Local\youdao\dict\Application\7.5.2.0\resultui\dict\?keyword=o-dichlorobenzene) | OG | OG | PG | I | I | I |
| chlorobenzene | PG | PG | PG | I | I | I |
| dichloromethane | OG | OG | PG | I | I | I |
| toluene | PG | TG | PG | I | TG | TG |
| o-xylene | PG | PG | PG | PG | TG | TG |
| ethylbenzene | I | I | I | PG | TG | TG |
| 1,3,5-trimethylbenzene | I | I | I | I | TG | TG |
| methanol | I | I | I | I | I | I |
| acetone | I | I | I | I | I | I |
| acetonitrile | I | I | I | I | I | I |
| THF | I | I | I | I | I | I |

Gel Concentration: 2.5 % (w/v). OG: opaque gel; TG: Transparent gel; P: precipitate; S: solution. I: Insoluble.

**Rheological Study**

**
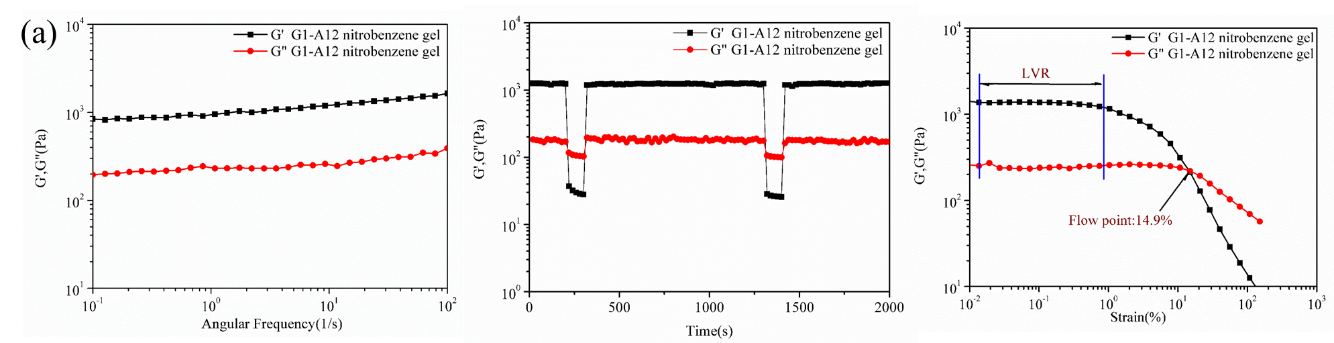
**

**
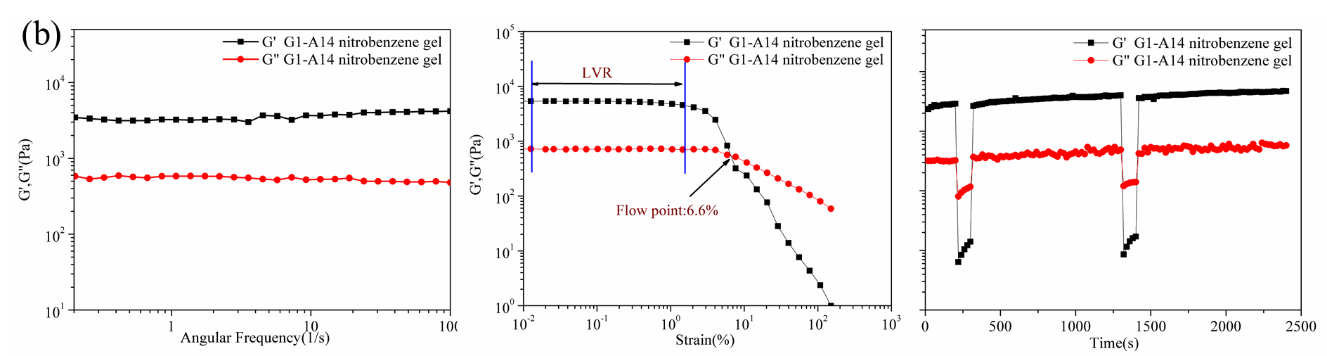
**

**
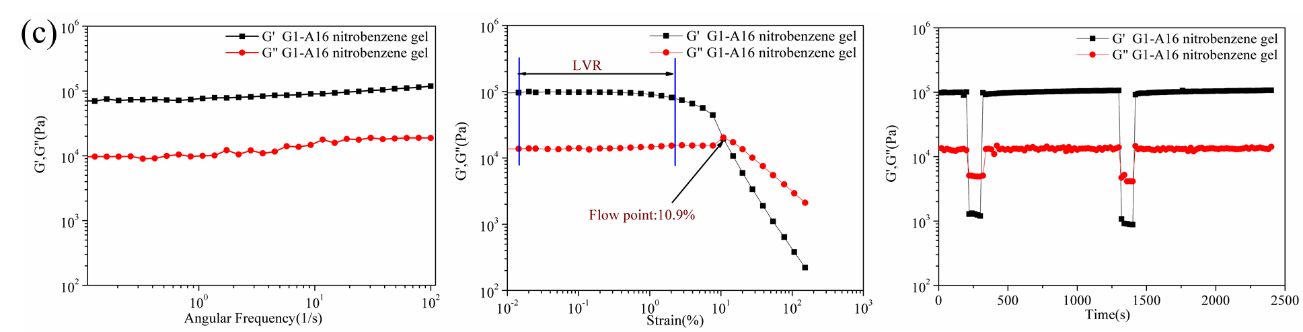
**


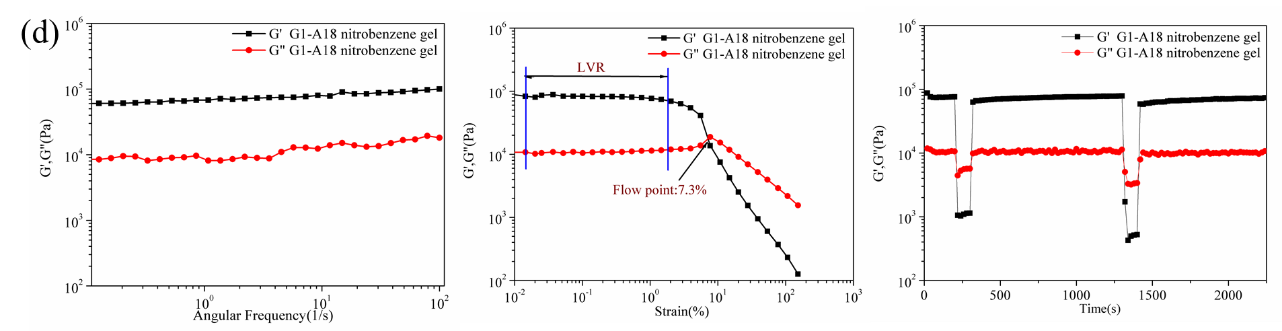


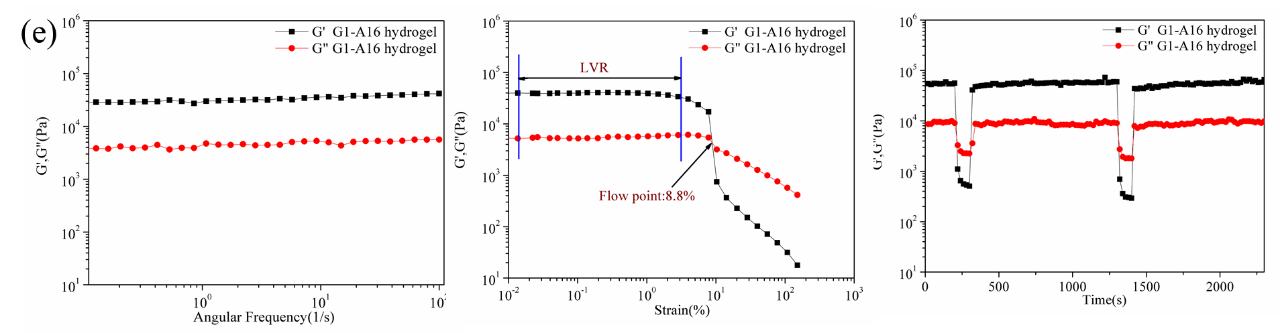


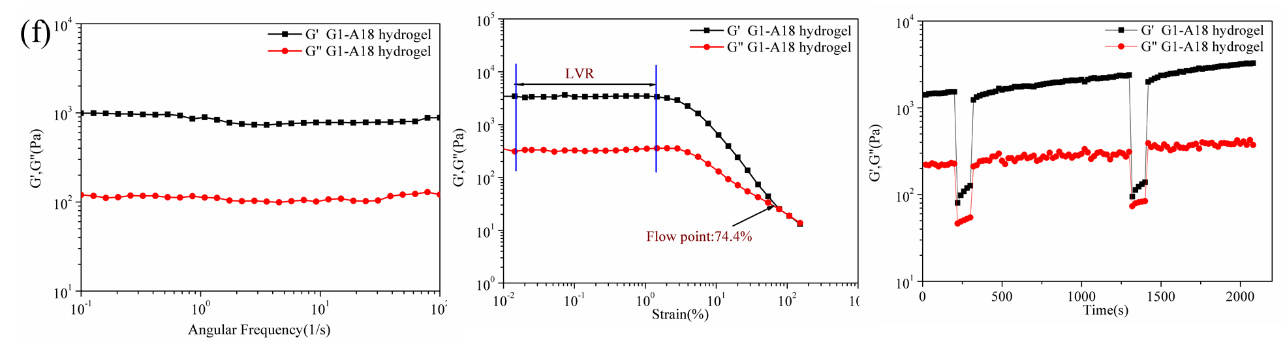


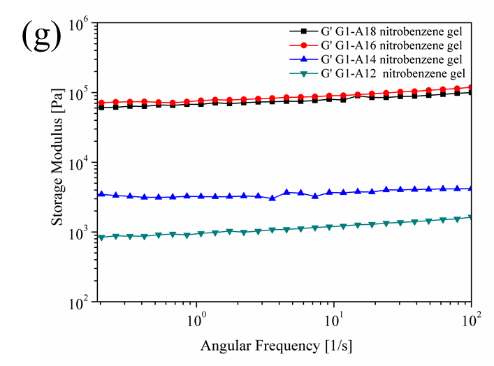


**Figure S1.** Oscillatory rheological study of gel from **G1-Am** (the molar ratio is 1:2, 2%, w/v) :(a) **G1-A12** nitrobenzene gel, (b) **G1-A14** nitrobenzene gel, (c) **G1-A16** nitrobenzene gel (d) **G1-A18** nitrobenzene gel (e) **G1-A16** hydrogel, (f) **G1-A16** hydrogel, (g) Frequency sweep of nitrobenzene gel from **G1-Am** with a fixed strain (0.1%) at 20℃.


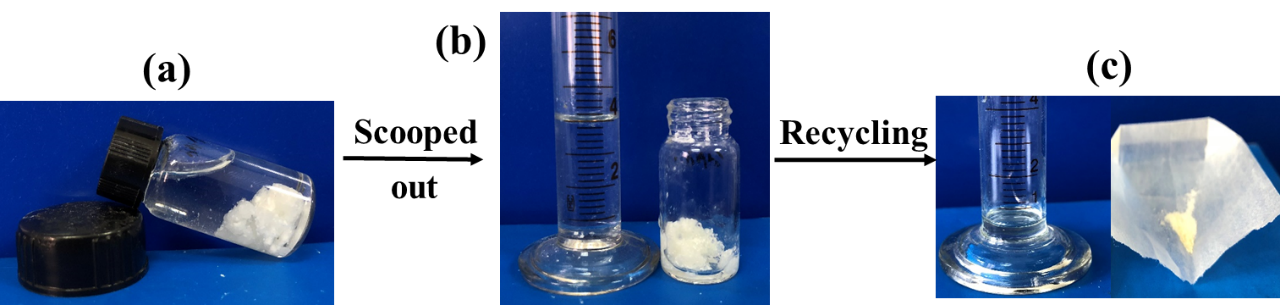


**Figure S2.** (a) Speciﬁc gelation of the o-dichlorobenzene phase using **G1-A16** (**G1-A16** is 40 mg) as a Phase selection gel in a two-phase mixture of o-dichlorobenzene and water (1 mL/3.0 mL，NaNO_3_ and Na_2_SO_4_ Concentration of wastewater is 0.5M) by mechanical shaking. (b) separation of the formed gel–water mixture into the o-dichlorobenzene gel via simple scooped out. (c)Recovery of o-dichlorobenzene from the **G1-A16** o-dichlorobenzene gel via distillation, and puriﬁcation of the restored gelator by recrystallization.

**Figure S3** The molecular structures of dyes

**Time-dependent UV-Vis spectroscopy measurement of G1-A16 xerogels-treated Methylene Blue aqueous solution.**


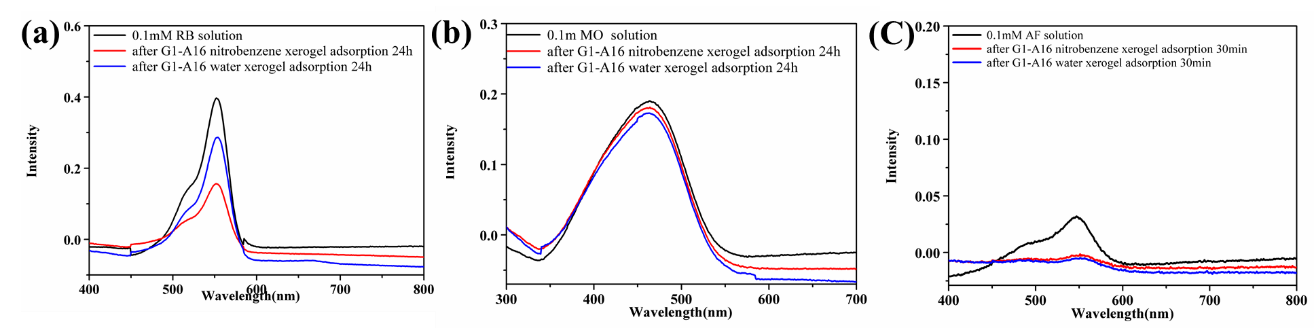


**Figure S4.** Time-dependent UV-Vis spectroscopy measurement of the **G1-A16** xerogels-treated **RB** aqueous solution (a), **MO** (b), (c) **AF**.

**Table S6** Maximum adsorption capacities of xerogels

| dyes | **G1-A16** nitrobenzene  xerogel (mg g^-1^) | **G1-A16** water  xerogel (mg g^-1^) | **B6-A16** water  xerogel (mg g^-1^) |
| --- | --- | --- | --- |
| AF | 610.75 | 594.09 | 17.2 |
| EY | 61.42 | 42.47 | 1.1 |
| MO | 1.64 | 2.95 | 2.15 |
| MG | 174.81 | 154.13 | 263.66 |
| MB | 32.31 | 31.92 | 168.8 |
| RB | 29.27 | 13.14 | 544.3 |

**References:**

[1] a) Xidong Guan, Kaiqi Fan, Tongyang Gao, Anping Ma, Bao Zhang and Jian Song. Chem. Commun., 2016, 52, 962-965; b) Shipeng Chen, Baohao Zhang, Nanxiang Zhang, Fengsheng Ge, Bao Zhang,Xiaoji Wang, and Jian Song, ACS Appl. Mater. Interfaces 2018, 10, 5871−5879.
